# Flow dynamics in rivers with riffle-pool morphology: a dataset from case studies and field experiments

**DOI:** 10.1038/s41597-023-02370-1

**Published:** 2023-07-27

**Authors:** Oleksandra O. Shumilova, Alexander N. Sukhodolov

**Affiliations:** Department of Ecohydrology and Biogeochemistry, Leibniz-Institute of Freshwater Ecology and Inland Fisheries, Berlin, 12587 Germany

**Keywords:** Freshwater ecology, Fluid dynamics, Hydrology

## Abstract

Bars and pools, common for natural riverbeds, form sequential patterns due to interactions between river flow, alluvium and vegetation. While the morphodynamics of bar-pool units are relatively well understood, far less is known about associated riffle-pool hydrodynamics because of a lack of high-resolution data collected in rivers and problems attaining natural scaling in laboratory studies. Here we present a dataset on turbulent flow structure in riffle-pool sequences of a natural river. Two case studies and two field-based experiments were carried out in a side branch of the braided gravel-bed Tagliamento River in Italy. Our case studies deliver detailed information about the three-dimensional structure of mean and turbulent flows in natural riffle-pool/run and pool-riffle/glide transitions. Field-based experiments completed with the in-stream flume models of a riffle-pool transition and a shallow jet model provide a methodological bridge for linking simplified hydrodynamic theories of shallow jets to complex flow structure documented by our case studies. Therefore, this dataset enables examination of scaling effects and can be widely used for validation of numerical models.

## Background & Summary

**Fig. 1:**
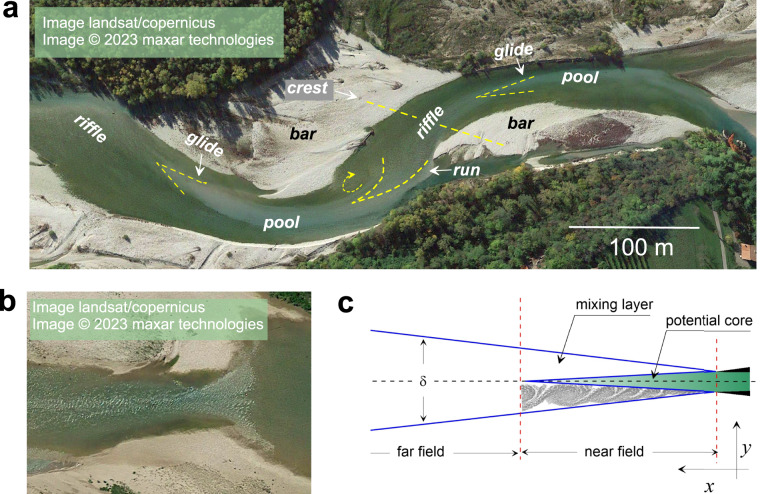
Background of the study design. (**a**) An example of a riffle-pool sequence in the Piave River, Italy (45°52′06″N 12°0′26″E). (**b**) Free surface pattern in a riffle-pool transition on the Tagliamento River, Italy (45°50′25″N 12°58′10″E), and (**c**) Schematic representation of a canonical jet flow (after Abramovich (2003)^29^), δ – jet width.

In rivers with alluvial riverbeds, the self-organized mechanisms have been found to control how small-scale perturbations on the riverbed grow into large-scale morphologic patterns or bedforms^1–4^. Bedforms are sequential topographic heights and lows in respect to the mean elevation of a riverbed and their longitudinal scale range from flow depth (*H*) to several flow widths (*B*)^4–8^. At intermediate/meso- scales^3^, bedforms are represented by alternate, braid, or point bars separated by topographical lows called *pools* (Fig. 1a). The line connecting the highest points of successive bars is the bedform’s crest, associated with the shallowest flow, and often is called a *riffle*^7,8^. Riffle-pool sequences provide vital ecological services to aquatic organisms and therefore are considered the fundamental habitats of invertebrate and fish assemblages in fluvial ecosystems^9–12^.

The fact that heights of bars scale with *H* and their wavelengths scale with *B* and *B*»*H* makes the simplified two-dimensional shallow-water equations an efficient hydrodynamic model in morphodynamical theory of bar stability^3,13^. This theory, advanced during the last several decades, reveals that riffle-pool sequence can evolve on initially flat riverbeds due to the destabilizing effect of bed friction (e.g. turbulence) while planar curvature forces the formation of stationary sequences of bars^3^. The theory predicts that the critical aspect ratio between sequence wave length and flow width ranges from 5 to 6 in agreement with reports of field and experimental observations^4,8,14^ and explains either a bar growths up-/down-stream or becomes stationary^3^.

Empirical geomorphological research of riffle-pool morphology dates back a century^4,5,8,14,15^. For the last five decades this research focused on the role that local hydraulics and sediment transport play for maintenance of riffle-pool sequences, which was conceptually driven by the velocity-reversal hypothesis^8,15^. Initially based on purely empirical comparison of near bed flow velocities at low and near bank full stages collected in a field case study^15^, the velocity-reversal hypothesis was later strengthened by one-dimensional hydraulic modelling to account for conservation of mass^16,17^. According to this concept, near bed velocities are greater in riffles at low water stage and then become smaller compared to that in pools at bank-full stage. Sediments are thus transported in pools at high flow events and deposited at riffles, while at low flow periods sediments are scoured from riffles and deposited in pools. Thompson (2011)^8^ presented a comprehensive review that concludes that the velocity reversal by itself does not fully explain riffle-pool maintenance. During high flow events, flow concentration (convergence of velocity vectors) and large velocity patches (jetting) are often reported in pools^17–20^. The importance of jet-like flow in the hydraulics of rapids, which are basically riffles impeding navigation, has been demonstrated by Kiefer (1989)^21^. For riffle-pool sequences it has been evidenced by laboratory experiments and two-dimensional numerical modelling^8,22^. Although three-dimensional turbulence-resolving measurements in the field are still scarce and limited to forced riffle-pools^19,23–26^, these studies provide strong evidence of the critical importance of jet-like dynamics.

Hydrodynamic theories of turbulent jets are fundamental in technical applications and have been recently advanced for shallow environmental flows^27–31^. Plane jets, most suitable for shallow flows^28^, are composed of two parts: potential core in the near-field and turbulent jet in the far-field (Fig. 1c). Potential core is a high-speed, low-turbulence fluid with a glossy surface separated from the ambient fluid by turbulent mixing layers. Similar patterns can be easily observed on the surface of river flows in riffle-pool and rapids^26,32^ (Fig. 1b). Jet theories offer tractable analytical solutions for mean velocity and turbulence profiles that are easy to use for processing of field data and obtaining integral parameters (e.g. total flow resistance) in non-uniform shallow flows^33^.

Visual patterns on the free surface of a river are often the primary sources of information in ecological sampling programs^9,10,34–39^, and recreational activities including fly-fishing and boating^39,40^. There is already consensus among ecologists on a qualitative understanding of basic features of water flow and free surface topography in riffle-pools. However, ecological classifications distinguish more elements in riffle-pool sequences of which runs and glides are most notorious^34,41^ (Fig. 1a), even though their definitions are often contradictive when examined using one-dimensional flow model^9,34,42^. At low flow, transition from a riffle into pool is accomplished by decelerating water moving on the back side of a bar as flow enters the pool (Fig. 1b). Transition from a pool into a riffle is accomplished with the acceleration of flow visualized by a “tongue”-shaped patch of fast gliding water, which is easy to spot at the free surface^32^ (Fig. 1a). At high flow events, the transition from riffle to pool can be observed further downstream that suggests the reversal in the jetting pattern as well^43^.

This state-of-the art review shows that previous and current research on morphodynamics, physical geography, fluvial hydraulics and stream ecology of riffle-pool sequences is heavily based on one- and two-dimensional approaches of hydraulics generically linked to the concepts of uniform-open channel flows^3,8,17,34,42^. Although there is growing evidence that flow structure in riffle-pool sequences is highly non-uniform and too complex to be tackled with conventional hydraulic approaches^4,9,20^, the lack of detailed case studies and specifically designed experimental work hinders applicability of hydrodynamic analysis based on the theory of jets^28,29^.

Here, we present a data set collected in the gravel-bed river Tagliamento in northeast Italy^44^. This data set is designed to advance knowledge of complex flow structures in riffle-pools by providing insight into three-dimensional hydrodynamics. Methodologically our study facilitates the dissemination of theoretical knowledge of jet hydrodynamics, which is often missing in applied research. The data set consists of two case studies and two controlled experiments in a naturally formed riffle-pool sequence. In our case studies, three-dimensional, turbulence-resolving measurements were completed in the transitions from riffle to pool (run) and in the pool to riffle transition (glide). Locations of cross-sections uniformly cover the areas of development of jet flows. Two field-based experiments were designed to obtain information on the effects of non-uniform bathymetry of the riverbed on the dynamics of a jet flow and to compare that to the dynamics of a shallow jet, which would develop on the same riverbed with uniform open-channel morphology. Because this research was performed on the same river reach, the data set provides methodological consistency with respect to skin friction roughness of the gravel riverbed while focusing on the effects of large scale bedforms. In addition to riverbed topography, the data set includes the results of detailed surveys of the free surface topography of the flow, information that is generally lacking in most field studies. We also demonstrate that spatial resolution of data resolves secondary flow circulations, which are critical for accurate parameterization of shallow jet dynamics by accounting for advective lateral exchange of momentum^33^. We believe that these data important for improving understanding of the physical mechanisms governing flow in riffle-pool sequences. It provides a pathway to better understand rarely-studied runs and glides and their connections to fundamental hydrodynamic theories. The data set can be used as a high-resolution supplementary data source for studies with application of eddy-resolving computational methods^45^.

## Methods

### Field research area and sites

Field data from case studies and field-based experiments were obtained at the Tagliamento River in northeast Italy near the town of Flagogna (46°12′9″N 12°58′15″E). The source of the Tagliamento River is in the Carnian Alps and the river flows about 178 km through pre-Alps and Friulian plains towards the Adriatic coast near Bibione-Lignano. The river’s hydrologic regime is flashy pluvio-nival with an average annual discharge of 90 m^3^s^−1^ near the study area^46^. The steep slopes of the Carnian Alps and torrential streams draining the upper catchment are the source of coarse bedload that is transported during floods and deposited in the central reach of the Tagliamento^47^. This river is believed to be the last intact river system in the Alps^46^.

**Fig. 2:**
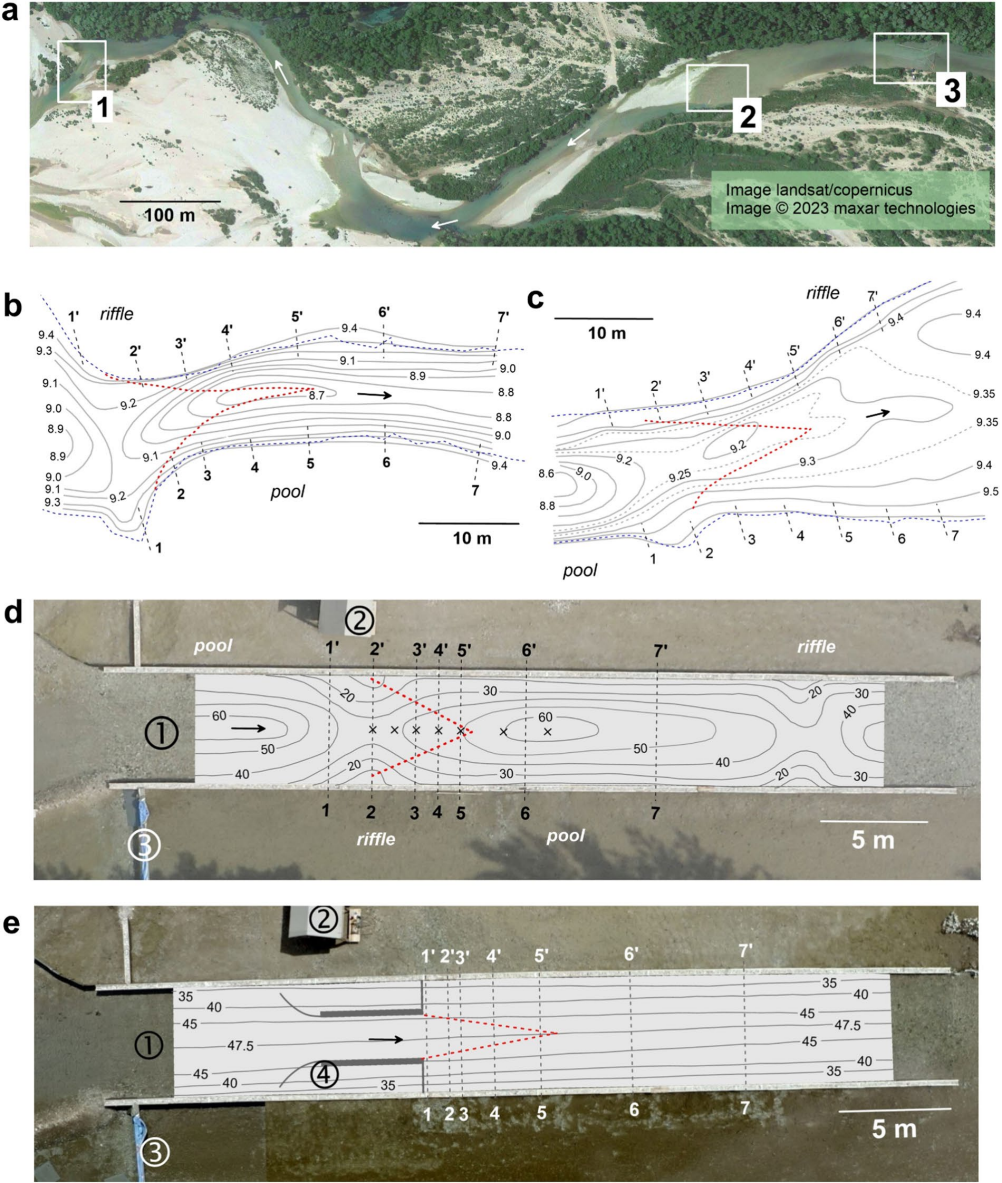
Research area and field sites. (**a**) Locations of field sites on the side branch of the Tagliamento River, flow is from the right to left (rectangles are enlarged in **b**–**e**). (**b,c**) topography of the riverbed in riffle-pool (1 in **a**) and pool-riffle (2 in **a**) sub-reaches, respectively (contour lines are elevations above arbitrary datum in m, blue dashed lines are contours of water edges). (**d,e**) bathymetry of riffle-pool sequence and shallow jet models, respectively, in the in-stream flume (3 in **a**, contour depth in cm, 1 = in-stream flume, 2 = operating platform, 3 = weir, 4 = jet nozzle). Red dashed lines in **b**–**e** show boundaries of visible potential cores of jet-like flow structures; black dashed lines are measuring cross-sections and arrows indicate flow directions. Symbols × (in **d**) indicate locations of multi-point vertical profiles.

The field research area is located on a right-side branch of the Tagliamento River and extends about 1 km upstream from the confluence with the Arzino River (Fig. 2a). This side branch provides a unique opportunity for carrying out field-based experiments on fluvial morphodynamics, hydrodynamics and ecology^33,48–53^. This is because it is characterized by a stable hydraulic regime during summer/mid-autumn seasons during which the perennial sources of the branch are separated by a gravel bar from the main river^52,53^. Three braid bars and pools form the main morphological units on this river reach (Fig. 2a). The riverbed material is gravel on average ranging from medium (*D*_16_ = 14 mm) to coarse (*D*_84_ = 38.7 mm) with *D*_50_ = 22.5 mm. At low flow during summer the mean discharge of the branch is about 2.5 m^3^/s. The channel width is about 20 to 30 m wide with depth ranging from 0.5 m in plain-bed and riffles to 1.5 m in pools.

The case study at the riffle-pool transition (CST-1) was carried out in May 2011 (location 1, Fig. 2a,b). Transition from pool to riffle (CST-2) was assessed in October 2010 (location 2, Fig. 2a,c), and field-based experiments (FE-3, FE-4) were completed in July-August 2021 (location 3, Fig. 2a,c,d). At CST-1 the riffle section is 21 m wide and 0.35 m deep, and the pool section is 12 m wide and 0.7 m deep (Fig. 2b). At CST-2 the pool section is 16 m wide and 0.8 m deep, while the riffle section is 29 m wide and 0.25 m deep (Fig. 2c). At the FE-3 and FE-4 location, the channel is a plain-bed^7^ section of the river reach (Fig. 2d,e).

### In-stream flume and models

The field-based experiments were carried out using the research platform RIVER-LAB of IGB, Berlin^33,53,54^. This methodological and technical platform is designed to form a methodological bridge to connect field and laboratory methods and a framework for a cross-disciplinary dialogue. The RIVER LAB relies on detailed instrumental measurements, which are performed under experimental control of variables in the in-stream flumes.

A 40 m long, 5 m wide rectangular in-stream flume was built in central part of the plain-bed section at the FE-3 and FE-4 location (Fig. 2a). At river left the channel was sealed by an impermeable wall and a weir was constructed on the right side (Fig. 2d,e). The rate of approach flow in the flume was controlled by adding or reducing the number of plastic plates in the weir. The side walls of the flume were assembled from 1.5 × 0.5 × 0.25 m gabions filled with the gravel. At the beginning of experiments the riverbed was flattened to obtain uniform depth of about 0.40 m and the riverbed material was used to make a funnel at the entrance to the flume.

Two flow models were created in the flume. The first one is the model of a shallow jet (FE-4), which was constructed on the flat riverbed by placing a nozzle with a 2 m wide outlet (Fig. 2e). The nozzle accelerates the flow to twice that of free flow velocity. The second model is the flow at a riffle-pool transition (FE-3, Fig. 2d). This model was created by excavating a pool and using the material to form a bar. The dimension of the cross-section at the bar crest was designed to be close to that of the nozzle. This produced acceleration of flow similar to that of the experiment with the shallow jet. These two experiments allow examination of how the dynamics of a shallow jet will change due to complex riverbed morphology characteristics of natural riffle-pool sequences.

### Field setup and instrumentation

**Fig. 3:**
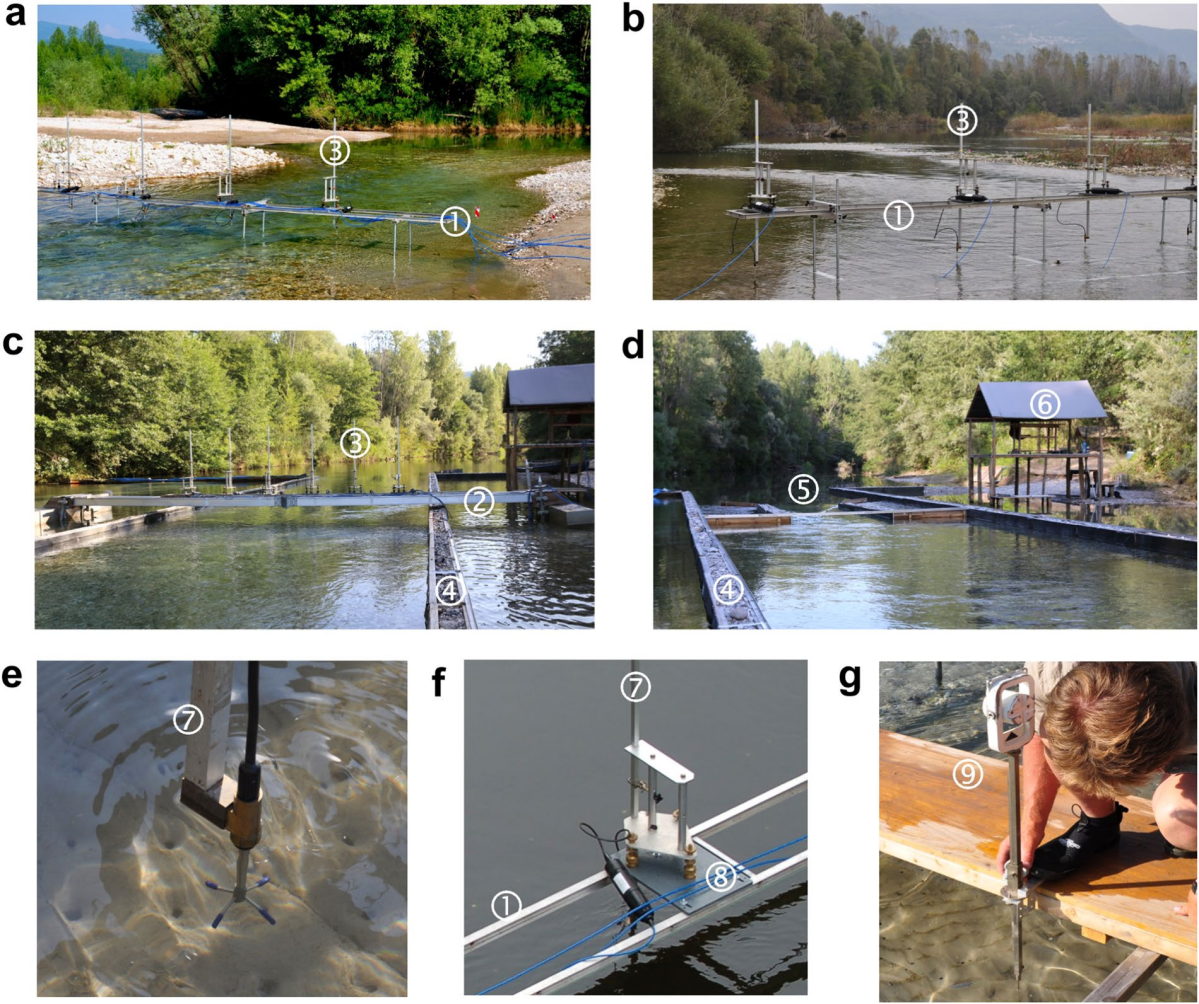
Experimental facilities and instrumental setups. (**a**–**c**) Instrumental setups of measurement studies in riffle-pool (**a**, Fig. 1a, 1) and pool-riffle (**b**, Fig. 1a, 2) sites and field-based experiments (**c**, Fig. 1a, 3), (**d**) nozzle in shallow jet experiment, (**e**) sensor head of a velocimeter mounted on a deployment road, (**f**) velocimeter mount, and (**g**) free surface survey pole (1 = flat frame platform, 2 = floatable platform, 3 = deployment mount, 4 = in-stream flume, 5 = jet forming nozzle, 6 = operating platform, 7 = velocimeter sensor attached to deployment rod, 8 = deployment mount with tribrach levelling mechanism, 9 = free surface gauge with a target prism).

In both measurement case studies we employed a custom-built lateral platform composed of a flat aluminium frame (Fig. 3a,b, item 1) supported by steel uprights. An array of five velocimeters was mounted on the frame using deployment mounts with tribrach levels (Fig. 3a,b, item 3). Velocimeters transducer sensors were fixed at the ends of deployment bars (Fig. 3e, item 7), which can be aligned vertically by levelling the mounts. The deployment bars were aligned horizontally along a line connecting the bench marks of cross-sections.

In the field-based experiments we employed a custom-built, floating lateral platform (Fig. 3c, item 2). The platform consists of a 9 m long flat aluminium frame suspended at both ends by two floats. The platform carried out an array of six velocimeters using the same deploying mounts from the case studies (Fig. 3c, item 3). The levelling and heading technique were the same as in the case studies.

Measurements of three-dimensional velocities were performed with acoustic Doppler Vectrino + velocimeters (Nortek AS, Norway) with a lab probe mounted on 1 m long cable (Fig. 3e). Velocimeters were connected by 30 m long cable to a portable computer at the operating platform (Fig. 3d, item 6).

Topography of the riverbed and free surface of flow was surveyed with an Elta 55 total station (Zeiss, Germany). In the case studies the reflector target was mounted on a laboratory needle gauge mounted on a moveable platform (Fig. 3g). Water levels were monitored at three gauging stations located in the entrance, middle and exit sections of field sites and models.

**Fig. 4:**
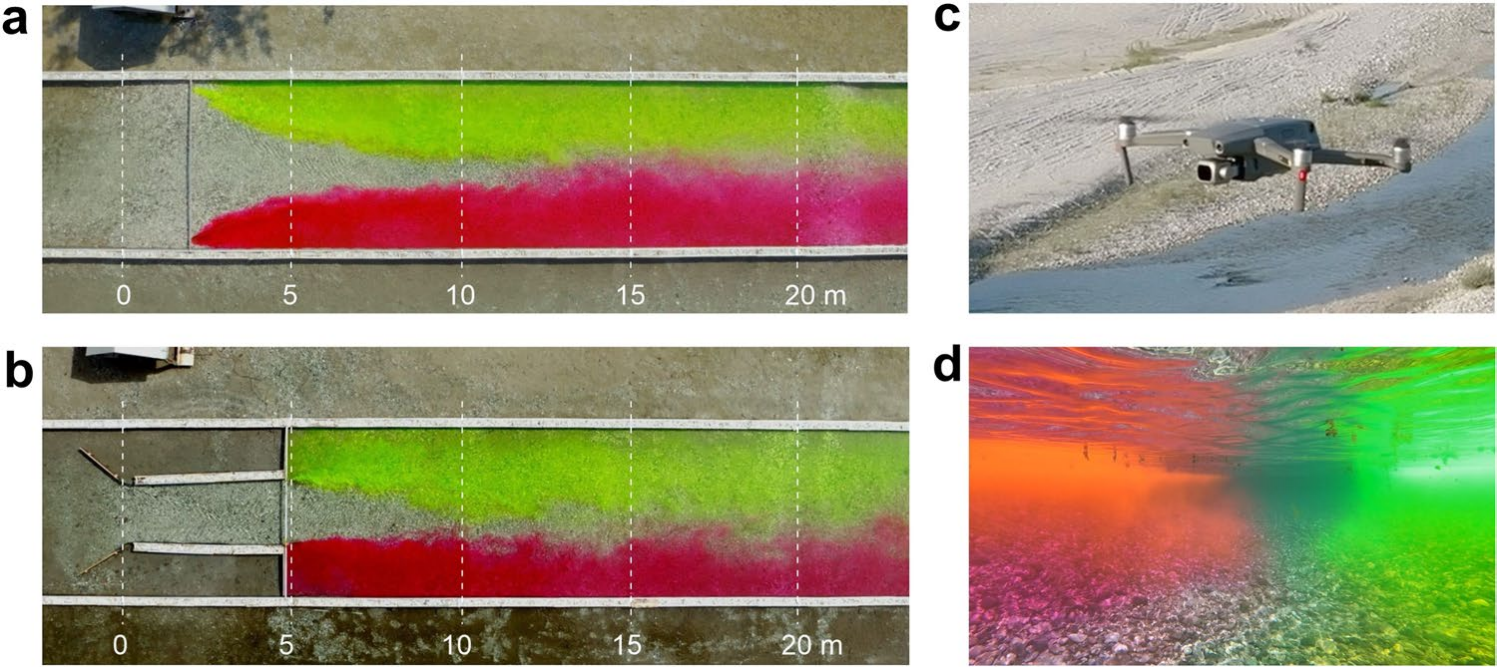
Flow visualizations in the field-based experiments. (**a,b**) Patterns of flow in the experiments with riffle-pool model and shallow jet, respectively (dashed lines indicate cross-sections marked on video records, (**c**) quadro-copter Mavic 2 PRO used in video recording of flow visualizations, (**d**) underwater video recording of flow visualization with a GoPro-9 action camera.

In the field-based experiments flow visualizations were conducted using continuous point injections of uranine and rhodamine fluorescent dyes (Fig. 4). A quadro-copter DJI Mavic 2 Pro was used to record visualization patterns in the fluid (Fig. 4c). Cross-sections were marked on the flume walls to aid in digital post-processing of remotely sensed images (Fig. 4a,b). Visualized flow patterns were also recorded underwater with a GoPro-9 camera (Fig. 4d).

### Measurement program of case studies

**Fig. 5:**
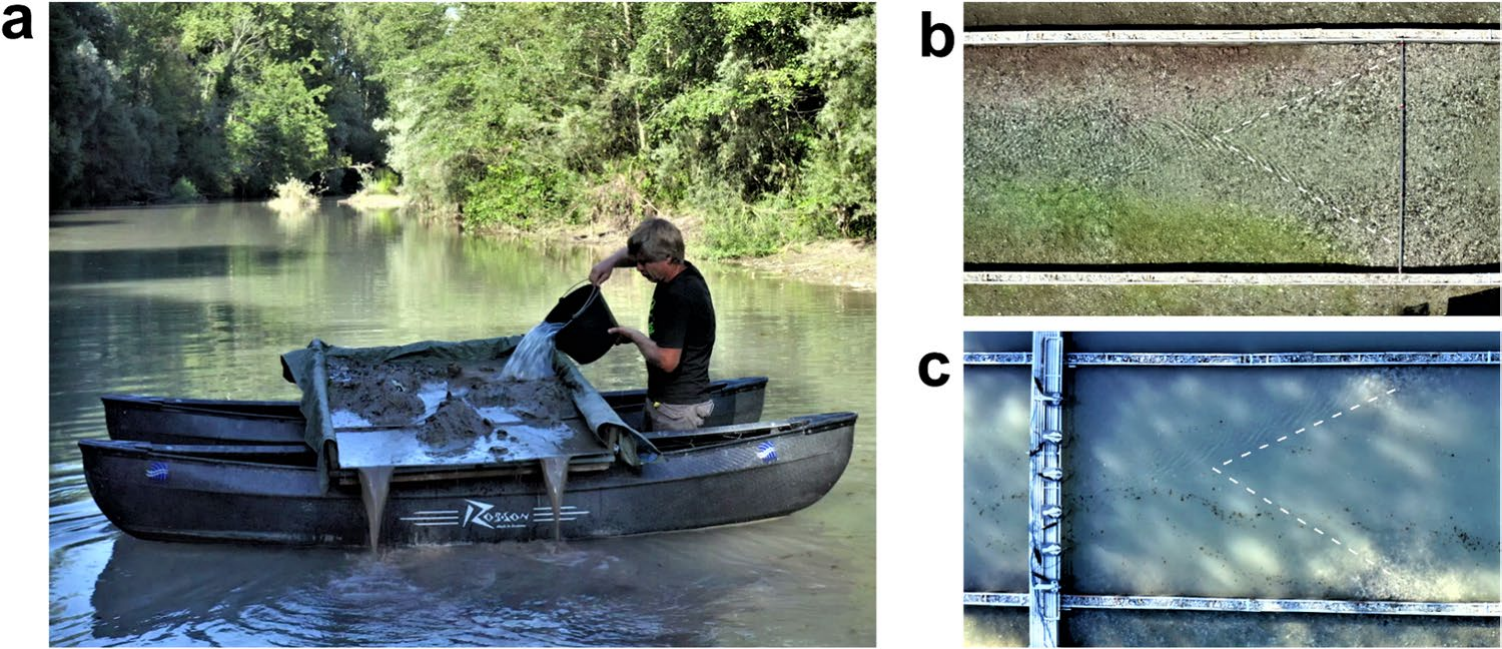
Flow seeding with fine suspended sediments. (**a**) Injecting sediment-water mixture from a floatable platform. (**b**) Flow without seeding material. (**c**) Flow after seeding the sediments. Dashed lines indicate the boundaries of near field of jet-like flow visualized by turbulent patterns.

The program of measurements was the same for both cases. Velocities were measured at 7 cross-sections aligned orthogonal to the local direction of the channel centreline (Fig. 2b,c). Measurements were performed at 10 verticals within each cross-section and at 5 points within each vertical. At each point velocities were sampled for 8 to 10 minutes at a sampling rate of 25 Hz. Because the river branch is fed by perennial groundwater flux the water was very clean and sampling with such high frequency required seeding of the flow with fine suspended sediments. This procedure was the same for case studies and field-based experiments (Fig. 5). Fine sediments deposited on the floodplain were loaded to the floatable platform, which was then positioned far upstream to provide optical spread of sediments across the flow. The sediments were mixed with water and spilled into the flow (Fig. 5a). The seeding therefore substantially increased the water turbidity and allowed for consistently high signal-to-noise ratios (Fig. 5b,c). We used about 150 kg of sediments, and the sediment cloud took about 10 minutes to pass completely through the measuring cross-section.

Topography of the riverbed and free surface measurements were performed before velocity measurements. Water levels were monitored during measurements by manually taking reading from the gauges every hour. Main hydraulic and morphological parameters of case studies are summarized in Table 1.

**Table 1:** Hydraulic and Morphological Parameters of Case Studies and Experiments.

LN	Study	*Q* m^3^s^−1^	*U* ms^−1^	*B* m	*H* m	Fr	Re ×10^−5^
*Case measurement studies*							
CST-1	Riffle-pool	2.53	0.78	9.61	0.34	0.43	2.23
CST-2	Pool-riffle	2.49	0.63	16.3	0.25	0.40	1.46
*Field-based experiments*							
FE-3	Riffle-Pool Model	0.55	0.28	5.0	0.39	0.15	1.07
FE-4	Shallow Jet Model	0.45	0.23	5.0	0.39	0.12	0.89

Note: LN is location name (Fig. 2b-e), Q is the discharge, *U* is the mean flow velocity, *B* is the mean flow width, *H* is mean flow depth, Fr is the Froude number, Re is the Reynolds number.

### Experimental program

Experiments with riffle-pool and shallow jet models were identical in their instrumentation and measurement protocols. They were completed during a one-months period when the hydraulic characteristics on the river reach were stable and water-level fluctuations were less than 1 cm. Velocity measurements were obtained in 7 cross-sections spaced more densely at the near field of the riffle-pool transition and shallow jet (Fig. 2d,e). At each cross-section velocities were measured at 12 sampling locations spaced laterally by 0.4 m intervals. Measurements were performed at the mid-depth points with sampling frequency of 25 Hz. Additionally, in the riffle-pool transition experiment a longitudinal profile through the centreline of the flow was measured at 7 verticals (Fig. 2d). At each vertical, velocities were measured at 12 points uniformly spaced over the flow depth with sampling frequency of 25 Hz. Measurements were supported with flow seeding and followed the same sampling scheme as that in the case studies. Main hydraulic and morphological characteristics of experiments are summarized in Table 1.

In addition to flow measurements, visualizations of flow patterns were performed during the experiments when the wind was minimal. Video records were obtained by positioning the drone stationary at the altitude of 40 m in GPS mode to compensate for wind-induced drifts. The duration of tracer injection was about 12 min and the video records were about 10 minutes long with a resolution of 3,040 × 2,160 pixels per frame at 25 frames per second in MP4 format.

The topography of the riverbed and free surface of the flow were measured with geodetical instruments similarly to the case studies. Water level readings were made manually at three gauges on hourly time intervals.

### Data postprocessing

The software package ExploreV (Nortek As, Norway) was used to post-process velocity time series collected in the field. Because of flow seeding during the recording time, each time series included time intervals with spurious spikes when the water was clean and after the sediment cloud have passed the measurement point. To extract the intervals when the flow was seeded, each time series was visually inspected and the intervals without seeding were removed by using the clipping capabilities of ExploreV. Single spikes that appeared in the intervals with seeding were removed and replaced with values generated by linear interpolation between adjacent data. Edited time series were further processed to calculate the statistical characteristics:

1 $${\overline{u}}_{i}=\mathop{\mathrm{lim}}\limits_{T\to \infty }\frac{1}{T}{\int }_{0}^{T}{u}_{i}\left(t\right)dt,\;i=1,2,3,$$

2 $${\sigma }_{i}^{2}=\mathop{\mathrm{lim}}\limits_{T\to \infty }\frac{1}{T}{\int }_{0}^{T}{\left({u}_{i}\left(t\right)-{\overline{u}}_{i}\right)}^{2}dt,$$

3 $$\overline{{u}_{i}^{{\prime} }{u}_{j}^{{\prime} }}=\mathop{\mathrm{lim}}\limits_{T\to \infty }\frac{1}{T}{\int }_{0}^{T}\left({u}_{i}\left(t\right)-{\overline{u}}_{i}\right)\left({u}_{j}\left(t\right)-{\overline{u}}_{j}\right)dt,\;j=1,2,3,$$

4 $$TKE=0.5{\sum }_{i=1}^{i=3}{\sigma }_{{}_{i}}^{2},$$

where *u* is velocity vector component, *σ*^2^ is velocity variance, $\overline{{u}_{i}^{{\prime} }{u}_{j}^{{\prime} }}$ is shear stress component, and *TKE* is turbulent kinetic energy. These statistical characteristics are the main information of our data set.

Topographical data were rendered to a local coordinate system and hand-contoured to obtain topographical maps of the riverbed (Fig. 2). These maps were produced manually because automated contouring software based on kriging interpolation algorithms could not accurately account for the spatial complexity with irregularly sampled data while also accounting for spatial variation of the free surface. We obtained the maps of the free surface using a similar manual process. These maps are included into supplementary section of our data set.

Post-processing of video records consisted of visual examination and selection of intervals of about 30 s long. The criteria for selecting an interval was that the pattern was fully developed, the tracer was distributed along the whole flume length and the heading of the camera remained the same in all frames (Fig. 4a,b). The video of selected intervals was then converted into a sequence of JPEG images (1,920 × 1,080 pixels) with the time span between images of 0.08 s. These images are stored in the data base for each experiment.

## Data Records

The data base accessible from Zenodo data repository is composed of the main folder named TGR_RP_DBASE, which contains two sub-folders named DB_CASE_ST (case studies) and DB_FB_EXP (field-based experiments)^44^. The sub-folder DB_CASE_ST contains two subfolders named accordingly with naming of studies in Table 1: CST_1 and CST_2. Each of these sub-folders contains further sub-folders named MAIN_DATA and SUP_DATA. The folder MAIN_DATA contains 7 Excel spreadsheets (*.xlsx), named CS-1, CS-2…CS-7. Each of these spreadsheets contains 11 pages: a summary page named after the cross-section name (e.g. CS-1, CS-2…CS-10) and pages corresponding to each of the measured vertical profile (e.g. P1, P2, P3 … P10). Naming of variables and their description is provided in Table 2. The summary page contains the name of the cross-section and numbers of vertical profiles according to the in-field measurement formula, where WE R and WE L is the water edge of right and left banks, respectively. The second column (L, m) contains distances from a bench mark in m, and the third (h, m) are depths in m. The SUP_DATA folder contains two files named RIVERBED_MAP.jpg and FREESURF_MAP.jpg, which are the riverbed map (elevations in meters above arbitrary datum) and a free surface topography (contours in cm). Both maps show positions of cross-sections. Additionally, the raw information is provided for the bench marks in the file BMARKS.xls and in the file CONTOURS.xls. The first contains coordinates x, y, z in meters for the bench marks located on the right (RB) and left (LB) banks, respectively. The second one provides coordinates x, y, z in meters for the water edges on both banks and for the border of jet potential core.

**Table 2:** Description of data variables.

nn	Variable name	Physical meaning	Equation	Unit
1	z	distance from a riverbed to the point		m
2	U	mean streamwise velocity	Eq. 1	cms^−1^
3	V	mean lateral velocity	Eq. 1	cms^−1^
4	W	mean vertical velocity	Eq. 1	cms^−1^
5	u’	streamwise velocity fluctuation, $\sqrt{{\sigma }_{1}^{2}}$	Eq. 2	cms^−1^
6	v’	lateral velocity fluctuation, $\sqrt{{\sigma }_{2}^{2}}$	Eq. 2	cms^−1^
7	w’	vertical velocity fluctuation, $\sqrt{{\sigma }_{3}^{2}}$	Eq. 2	cms^−1^
8	u’v’	lateral turbulent flux of momentum	Eq. 3	cm^2^s^−2^
9	u’w’	vertical turbulent flux of momentum	Eq. 3	cm^2^s^−2^
10	v’w’	longitudinal turbulent flux of momentum	Eq. 3	cm^2^s^−2^
11	k	turbulent kinetic energy (TKE)	Eq. 4	cm^2^s^−2^

The sub-folder DB_FB_EXP contains two sub-folders named accordingly to experiments as in Table 1: FE_3 and FE_4. Similar to the case studies, each of these sub-folders contain sub-folders named MAIN_DATA and SUP_DATA. The folder MAIN_DATA contains 7 Excel spreadsheets (*.xlsx), named CS-1, CS-2…CS-7 corresponding to measurements performed at mid-depth in each cross-section in the flume (Fig. 2d,e). The variables in these spreadsheets are the same as defined in Table 2. In the folder FE_3, the additional spreadsheet LONG_PROF.xls contains vertical profile data measured at the longitudinal profile along the centreline of the flume (Fig. 2d). Each page in this spreadsheet is named P1, P2…P7 and indicates the number of the vertical profile in a sequence starting from the riffle section. Each of the pages contains measurements in the format shown in Table 2. In addition, the sheets contain the lateral offset of measurement location from the left wall of the in-stream flume (Fig. 2d,e). The sub-folder SUP_DATA also includes two files MODEL_BED.jpg and FREESURF_MAP.jpg, which represent bathymetry (depth contours in cm) and free surface topography (contours in cm). The folder SUP_DATA contains also a sub-folder VISUAL where sequences of images obtained from flow visualization are stored. This folder also contains an Excel spreadsheet file named BMARKS.xls, which provides x, y, z coordinates for cross-sections shown in Fig. 4a,b. The coordinates are given for the external side of the flume at the left bank and for the internal side at the right bank. These points can be identified on the images and used for digitalization. In addition, the folder SUP_DATA contains an Excel spreadsheet CONTOURS.xls with coordinates of flume walls, nozzle (for FE_4) and jet potential core.

## Technical Validation

The assessment of data validity of our study involves analysis of initial data quality, analysis of data resolution with respect to main flow features, and assessment of accuracy of the supportive data. The main output data of our experiments were time series of flow velocity and their technical validation required application of time-series analysis. Analysis of data resolution involves examination of important flow features that can be expected based on comparison with previous research. The supportive data rely on geodetic and hydrometric measurement that require assessment of accuracy in our field studies.

### Time-series analysis of velocity measurements

**Fig. 6:**
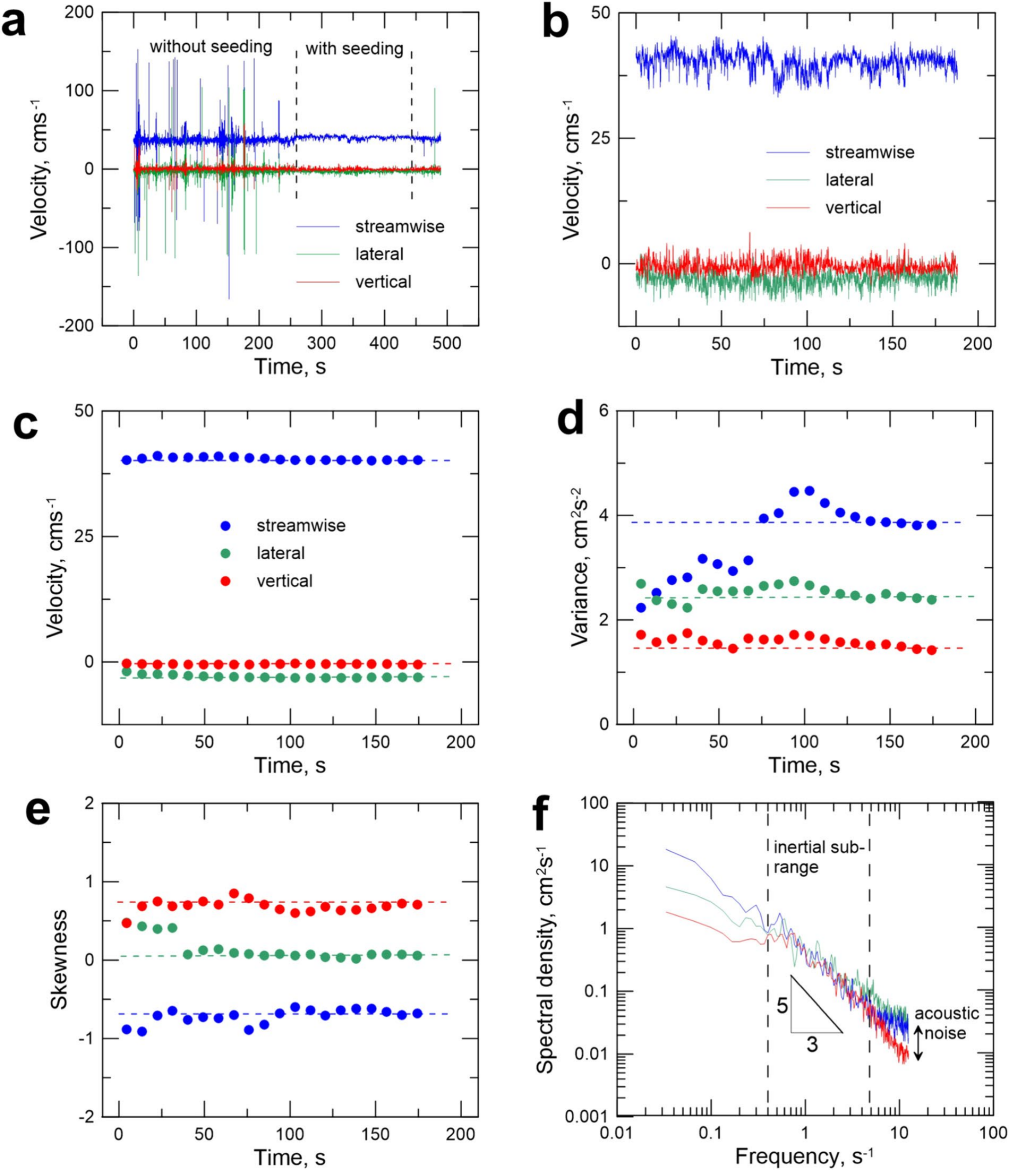
Time-series analysis of measured velocities. (**a**) Example of raw data measured in the field-based experiments (dashed vertical lines indicated boundaries of the clipped time-interval). (**b**) Edited time-series. (**c**) Cumulative mean of time-series. (**d**) Cumulative variance of time-series. (**e**) Cumulative skewness of time-series. (**f**) Spectral density of time-series.

Analysis of three-dimensional velocity time-series measured in turbulent flow was performed for each of measured time-series in this data set with the use of ExploreV software package. This analysis includes time-series editing, testing of stationarity and spectral analysis. Time-series editing started with visual inspection of raw data records and was aimed at detecting time intervals during which the flow was seeded enough to produce high quality data. Figure 6a shows a typical time-series sampled for about 9 min. The velocity records without seeding contained many spurious spikes. By clipping this time-series we obtain about 3 minutes of high-quality record. Stationarity of the times-series was assessed by dividing the retained high quality interval into 20 equally spaced sub-intervals and then measuring a running mean, variance and skewness for these 20 sub-intervals (Fig. 6c–e). If the data are stationary, the cumulative statistics gradually should converge on a constant value as the number of sub-intervals included in their computation increases. The example presented here illustrates that after about 120 s all statistics converge. Relatively accurate results can be obtained even at about 60 s sampling periods, though to obtain statistics relevant to accurately estimate velocity profiles a period of about 180 s is preferable^55^. Except in a few cases, most of the recorded data conformed with these criteria because of the visual quality control of data acquisition in the field. The next step in the analysis was the spectral analyses of time-series. Turbulence spectra were obtained by applying a Fourier transformation of the covariance function and smoothing obtained periodograms of spectral density with Parzen’s correlation window^56^, Fig. 6f. Comparison of spectra for different components allows estimation of the cumulative effects of measurement errors due to the finite sampling volume of velocimeters. Because the vertical component is measured with higher accuracy than the horizonal components, by integrating spectra at high frequencies and subtracting obtained values of the vertical component we estimate that the horizontal components were measured with an average accuracy differing about 2% from the true value. In almost all data we also had a good separation between acoustic noise level and low frequency spectral density ensuring high signal to noise ratios.

### Spatial resolution analysis

**Fig. 7:**
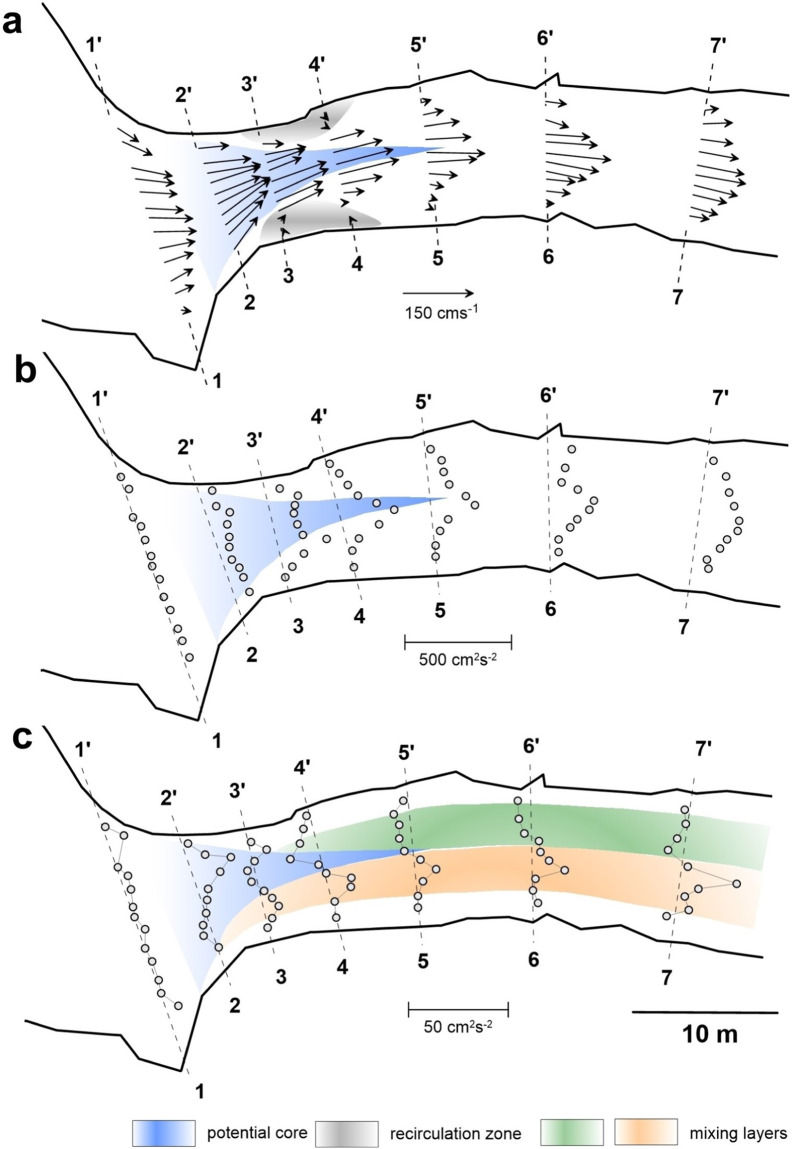
Spatial patterns of depth-averaged flow characteristics in a riffle-pool transition, CST-1. (**a**) Velocity vectors. (**b**) Turbulent kinetic energy (TKE). (**c**) Lateral turbulent fluxes of momentum $-\overline{u{\prime} v{\prime} }$.

**Fig. 8:**
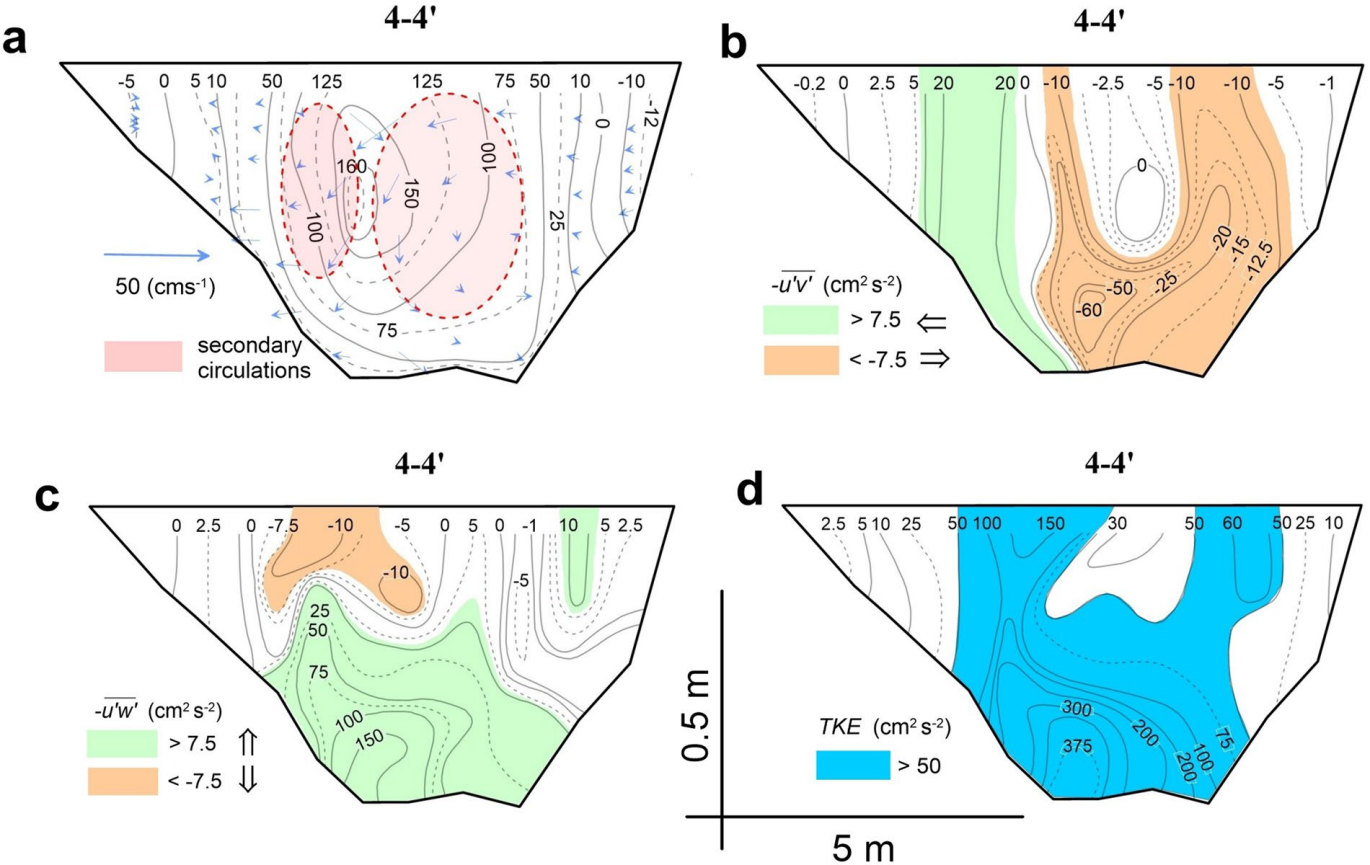
Cross-sectional patterns of measured flow characteristics in a cross-section 4-4′, CST-1. (**a**) Patterns of mean streamwise velocities (cms^−1^) and cross-stream velocity vectors (ellipses outline secondary circulation cells). (**b**) Patterns of lateral turbulent fluxes of momentum $-\overline{u{\prime} v{\prime} }$ (colours indicate directions). (**c**) Patterns of vertical turbulent fluxes of momentum $-\overline{u{\prime} w{\prime} }$ (colours indicate directions). (**d**) Patterns of TKE.

Because this study focuses on spatially varying flows, the question of spatial data resolution is of key importance. Our case studies and experiments are characterized by similar flow structures, and in this section we illustrate our analysis on example of CST-1. In general, the three-dimensional flow evolves over considerable distances that are also characterized by high spatial variability of riverbed morphology (Fig. 2). Observations of the free surface in the field allowed us to define the zones of jet potential core bounded by mixing/shear layers, flow recirculation zones, and recovery zones. Locations of measurement cross-sections and vertical profiles were guided by these observations and allowed us to resolve these features, which are evident in depth-averaged flow patterns (Fig. 7). Patterns of lateral turbulent fluxes of momentum allow us clearly differentiate between differently directed turbulent motions and outline the boundaries of mixing layers evolving on the sides of jet-like flow in the middle (Fig. 7c). Without knowledge of these fluxes, determination of zones based on mean velocity patterns and turbulent kinetic energy, an integral characteristic, would be difficult (Fig. 7b). Because of the strong three-dimensionality of flow, the dataset for these case studies also provides detailed information on time-averaged and turbulent flow structures in the cross-sections (Fig. 8).

The example of cross-section 4-4′ shows that several distinctive features of flow structure were resolved by our measurements. First, the measured data clearly demonstrate the descent in the location of velocity maximum, so-called “velocity-dip” phenomenon^57^, from the free surface towards the riverbed (Fig. 8a). This is also accomplished by development of two counter-rotating cells of secondary circulation, which are bounded on the sides by the flow recirculation zones. The zones of strong shear between recirculation zones and the jet core are characterized by vertical strips of enlarged values of lateral turbulent fluxes of momentum with opposing signs (Fig. 8b). Vertical turbulent fluxes of momentum indicate an area of high values near the riverbed due to friction of high velocity jet core with the riverbed (Fig. 8c). Near the free surface of the flow there is a patch of enlarged turbulent fluxes of momentum with downward motion of fluid, which is indicative of the downward descend of the potential core. This is an important feature that makes the flow in potential core smooth at the surface. Resolving these features with only turbulent kinetic energy would not allow partitioning the contribution of different structures.

In the field-based experiments EF-3 and EF-4 the focus was set on the horizontal structure of flow related to shallow jet models. Therefore, we increased the resolution in the lateral direction by reducing the distance between vertical profiles to 0.4 m and thereby resolving the structure scaling on the flow depth. In the experiments with riffle-pool model the vertical resolution of profiles was increased up to two times. Thereby the resolution of the experimental data also ensures that the flow structures of interest are resolved properly.

**Fig. 9:**
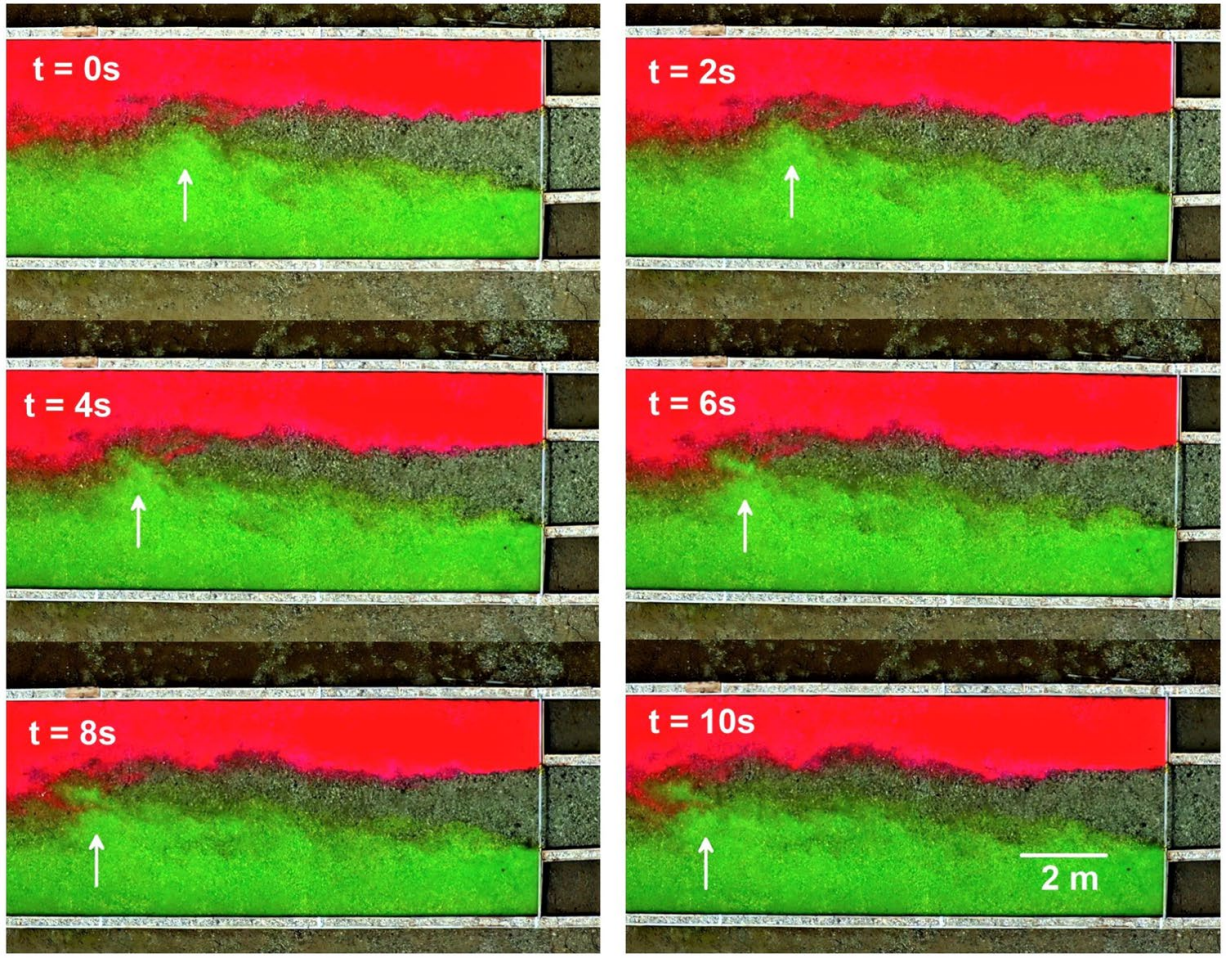
Sequence of flow visualization patterns obtained in the experiment with shallow jet model, FE-4 (flow is from right to left, the arrow points to a position of an example vortex).

In addition to measurements of flow velocities, in the field-based experiments we also resolved turbulence by visualizing the flows (Fig. 4). Because visualization experiments were completed with a high temporal resolution of 25 Hz, the information obtained by this method resolves flow structures similar in temporal scale to the three-dimensional in-stream velocity measurements. An example of this is illustrated in Fig. 9, which shows that individual turbulent structures can be distinguished at the scales about two times smaller than flow depth. This data provides the possibility to explore the dynamics of coherent structures on the Lagrangian frame of reference and to compare them with velocity measurements obtained with an Eulerian perspective.

### Supportive data

Supportive measurements in our research consist of geodetic surveys. The accuracy of these surveys was increased by using short-range measurements. Most of the measurements were completed within 30 m distance from the total station to the target, and vertical positions were always adjusted with the bubble level. In field case studies the riverbed was surveyed directly by wading and setting the target in multiple cross-sections and many points across the river. In field-based experiments bathymetry was measured in cross-sections spaced 1 m apart within the length of the flume. At each cross-section, depth measurements were performed at 0.5 m intervals with a sounding rod. The accuracy of depth readings was within 0.5 cm. The riverbed topography and bathymetry in all cases is shown in Fig. 2.

**Fig. 10:**
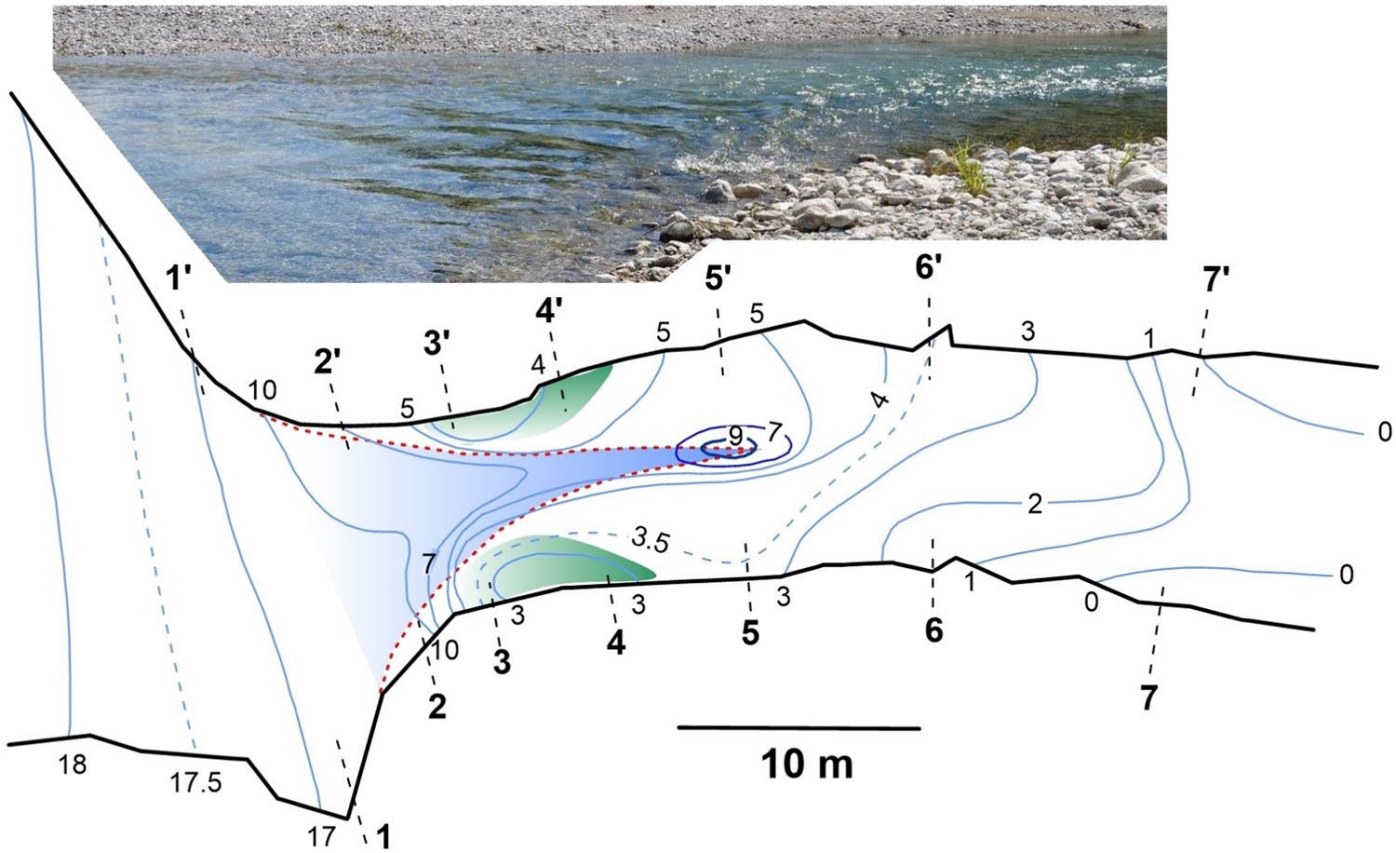
Topography of the free-surface, CST-1 (free surface contours in mm). Contour lines are uneven and reflect water surface elevation, which is not uniform within the sampled reach.

Free surface topography was measured with a total station reflector target mounted on a gauge with a sharp end, which was manually adjusted to an average elevation of the free surface at a point. Before a reading with the total station was performed, the gauge was adjusted roughly during a 1-min time interval to account for small local variations due to turbulence. Our estimate of accuracy in the free surface reading is about 1 mm at a smooth surface and about 3 mm at wavy zones at the boundaries of mixing layers. Sampling of free surface topography within cross-sections was more intense in the zones where changes in elevation where detected visually (e.g. end of a jet potential core). An example of free surface topography is shown in Fig. 10. It shows that there is a considerable difference in free surface between the upstream and downstream pool of the sequence. Measurements allowed us to resolve the low head zones in the recirculation zones and high pressure zone at the apex of the potential core. The magnitudes of these features are significantly larger than the accuracy of the measurements, thereby confirming both the sufficient accuracy and resolution of obtained data.

## Usage Notes

This data set provides high resolution data on the hydrodynamic structure of flow in riffle-pool sequences on a natural river supported with field-based experiments examining jet dynamics in the same riverine environments. A combination of these two methodological approaches allow bridging a gap between idealized hydrodynamic theories and complex natural processes. Therefore we believe that the data set can be used for generalization of field, laboratory and numerical research with the help of analytical solutions offered by hydrodynamic theories. Furthermore, because of the availability of detailed information on bathymetry and free surface topography the data set can be used for validation of models developed in computational fluid dynamics.

## Data Availability

No specific code has been developed for this study.
